# Supplementary material for: Are carbon emissions trading and green financial instruments synergistic? -Comprehensive quantitative research based on content analysis
Source: PLoS One. 2024 Mar 7;19(3):e0298601. doi: 10.1371/journal.pone.0298601 (PMC10919601; doi:10.1371/journal.pone.0298601)
Supplement: S1 Data — (ZIP) [file pone.0298601.s001.zip › Data/地级市Z_F工作报告.zip!/║■▒▒/╧╠─■╩╨/└┤╫╘╣1⁄2╓┌║┼í╛┬φ┐╦╩2╛▌═°í┐_2018.docx]

各位代表：

现在，我代表市人民政府向大会报告工作，请予审议，并请各位市政协委员和列席人员提出意见。

2017年是本届政府履职的起步之年。一年来，在省委、省政府和市委的坚强领导下，市政府团结带领全市人民，以习近平新时代中国特色社会主义思想为引领，按照省第十一次党代会确立的新定位新目标和市第五次党代会的总体部署，坚定不移地践行新发展理念，大力实施“创新驱动、绿色崛起”战略，砥砺奋进，求真务实，推动了经济社会持续健康发展，较好完成了全年目标任务。

——经济发展稳中有进。预计全年实现地区生产总值1205亿元，增长8%；规模以上工业增加值增长8%；社会消费品零售总额496亿元，增长12%；一般公共预算收入87.7亿元，可比增长9%左右；出口总额22亿元，增长15%。经济发展呈现“稳中有进、稳中向好、稳中显优”的良好态势。

——项目投资气势上扬。预计全市固定资产投资1648亿元，增长14.5%。其中，民间投资增长16%，增幅居全省前列。通过举办重大项目集中开工活动，新开工项目360个，总投资1889亿元。省级领导督办项目、省级重点项目和市级重点项目超额完成年度目标任务。

——发展质效持续提升。高新技术产业增加值增长12%，占工业增加值比重提高到22%。规模以上工业企业利润总额增长6%，税收占一般公共财政收入比重同比提升3个百分点。新增市场主体和注册资本金分别增长6.1%、46.8%。三次产业结构调整为16：48：36，服务业增加值对经济增长贡献率上升到50%。全员劳动生产率达到7.4万元/人。

——改革创新重点突破。实施112项重大改革，14项国家试点、18项省级试点初见成效，改革红利进一步释放。咸宁高新区成功晋级国家级高新区，瀛通通讯公司实现咸宁企业主板上市“零”的突破，成功获批首批国家农业可持续发展试验示范区，财政管理绩效考核受到国务院表彰。

——人民生活不断改善。民生支出占一般公共预算支出的比例超过75%。预计城镇和农村常住居民人均可支配收入分别达到28110元、13965元，分别增长8.8%、9%，跑赢GDP增速。居民消费品价格指数上涨1.8%。脱贫7.2万人、贫困村出列64个、易地扶贫搬迁1.54万人。群众衣食住行、业教保医等得到进一步保障。政府承诺的十件实事基本完成。

一年来，我们主要做了以下工作：

（一）聚力“三抓一优”夯实发展基础。全面落实市委“三抓一优”决策部署，开展“一季一拉练、一季一排名、一季一开工、一季一签约”等系列活动，有力推动了项目建设。投资过10亿元的咸宁南玻光电、咸安多肽原料药、嘉鱼华夏幸福新城、赤壁维达力二期、通城宝塔光电、崇阳柃蜜小镇、通山南林工业园等项目加快建设。落地PPP项目14个，总投资195.8亿元。建立覆盖市县两级的项目储备库和建设库，投资总额分别达到3780亿元、1633亿元。把招商引资作为头等大事，出台招商引资“17条”，全市新签约项目890个，协议投资总额2421.6亿元，到位资金1057亿元，引进中船重工等一批国内外500强企业。落实市委“新七条”，推进“互联网+政务服务”，开展9期电视问政，出台工业项目“零”审批方案，发展环境明显优化。

（二）聚力“三篇文章”推动产业升级。全力落实市委五届二次全会精神，做好高新技术产业、现代农业、全域旅游“三篇文章”。以高新技术产业助推工业升级。新增高新技术企业66家，总数达到 192 家，高新技术产业增加值占GDP比重达到9.6%。积极推进“一工程三计划”，新增规上工业企业89家，总数达到880家。智能机电产业入选国家创新型产业集群试点，省级重点成长型产业集群达到11个。以打造“名、特、优”产业助推现代农业发展。农产品加工业产值达到895亿元，与农业总产值之比为2.5:1。建成特色农产品基地770万亩，“赤壁青砖茶”“通山枇杷”入选省级特色农产品优势区项目。建成6家绿色有机农产品示范基地，“三品一标”达到425个。建成“一镇一业”专业镇11个，赤壁赵李桥镇、通山大畈镇、崇阳铜钟乡入选全省首批农村三产融合示范乡镇。以全域旅游助推现代服务业发展。加快国家级旅游业改革创新先行区建设，旅游人数、旅游总收入增速均保持在20%以上。文化消费不断扩大，书报杂志、体育娱乐类增长超过20%。出台大健康产业发展政策，江南里医养小镇等项目快速推进。实施商贸企业壮大工程，新增限上企业35 家。全市电子商务交易额131亿元，增长31%。贺胜金融小镇入驻基金公司158家，管理资金过300亿元。

（三）聚力改革开放增强创新活力。坚持改革创新开放融合推进，着力培育发展新动能。有序推进各项改革。深入推进供给侧结构性改革，化解煤炭过剩产能6万吨，关停烟花爆竹企业5家，商品房去库存消化周期7.7个月，降低企业成本9.35亿元。大力推进“放管服”改革，市级行政事业性收费项目、政府定价管理的经营服务性收费项目分别精简到15项、10项。稳步推进行政管理体制改革，市直部门“大科室”制改革基本完成，农业领域“四集中”改革走在全省前列。加快推进重点领域改革，监察体制、城管执法体制、乡镇财政体制、市区教育体制、社会保险制度等改革取得积极进展。加快实现动能转换。实施创新驱动战略，建成创新孵化平台18个，省级工程技术研究中心达到8家。实施招才引智工程，引进国家“千人计划”2人、海内外高层次人才163名，与北京院士专家开展合作。实施金融领先战略，成立绿色产业发展投资有限公司，发挥城发集团、高新投资集团、金融投资集团等平台作用，推动金融产品无缝对接企业，支持实体经济发展。金融机构存贷款余额分别增长15.5%、17.6%，贷存比61%。积极扩大对外开放。深度推进“走出去和引进来”系列活动，机电产品出口同比增长45.5%，23家企业实现出口零的突破。积极参加第三届楚商大会、2017丝绸之路国际博览会和鄂港澳经贸洽谈等活动，全力承办全国台企联片长联席会，与澳大利亚伊普斯维奇市、马来西亚诗巫市和纳闽市签订友城合作协议，推动了外向型经济发展。

（四）聚力生态保护提升环境质量。始终坚持生态优先、绿色发展，坚决保护好咸宁的绿水青山。实施“蓝天、碧水、净土”工程。市城区空气质量优良率稳中有升，PM10平均浓度值下降16.9%。纳入省考跨界断面和城市集中式饮用水水源地水质全部达标。启动土壤污染核实工作，确定详查点位932个。加强突出环境问题整改。87个突出环境问题全部销号，56件中央环保督察组信访交办件按时办结，38项中央环保督察反馈意见整改达到序时进度要求，现有黄标车全部淘汰。实施长江大保护十大行动，清理整治沿江化工企业51家，取缔关停长江干线非法码头37个，关停关闭非煤矿山109家，关闭规模养殖场72家，拆除湖库围栏围网18.7万亩。推进美丽咸宁建设。完成造林52.2万亩，创建国家级生态村2个、省级生态乡镇24个和生态村250个。成功加入全球可持续发展城市标准联盟，顺利通过全国水生态文明城市建设试点验收。全面建立河湖库长制，大力推广绿色建筑，加快整治环境卫生，逐步规范农村建房。

（五）聚力城乡统筹推进协调发展。实施区域协调发展战略，加快推进城乡融合发展。交通条件明显改善。完成交通基础设施投资54.6亿元，嘉鱼长江大桥、武深高速嘉鱼北段等一批重大项目快速推进。幕阜山旅游公路基本建成通车。新改建农村公路620.2公里，整治交通安全隐患800处，完成安全生命防护工程3602公里。市县城区内涵发展。编制完成《咸宁市城市发展战略规划》，构建了“一城十区二十景”发展新空间。市城区中心花坛改造全面完成，永安阁景区初具雏形，淦河大桥建成通车。城市防洪排涝整治、地下综合管廊、污水处理和污泥处置等工程加快推进。县级城区质量不断提升，功能不断完善。全市城镇化率达到52. 4%。镇村建设步伐加快。咸嘉生态文化城镇带和幕阜山绿色产业带建设成效明显。获批国家重点镇11个，省级重点镇7个。嘉鱼官桥镇入选全国特色小镇，咸安贺胜桥镇入选全省特色小镇，通山板桥村入选全国文明村。实施“两治两改一建”专项行动，新建村邮站200个，行政村全部通光纤。

（六）聚力民生改善促进社会稳定。坚持以人民为中心的发展思想，不断在发展中保障和改善民生。强力推进精准脱贫，率先在全省实行市级统筹购买精准扶贫补充医疗保险，是全省唯一实现 “医保一票制”即时结算的地区。新增城镇就业4.8万人，城镇登记失业率2.1%。城乡居民养老保险综合参保率98.8%，低保、五保户保障标准进一步提高。共发放社会救助资金4.9亿元，救助困难群众23.7万人次。全部按新标准发放高龄津贴。基本建成各类保障性住房12605套，棚户区改造开工11219套。全市新建、改扩建学校138所，获批全国首批现代教育装备技术综合改革实验区。取消公立医院药品加成，通过国家卫生城市复核审查，荣获全国创建幸福家庭活动示范城市。成功举办第九届国际温泉文化旅游节，“咸马”关注度再创新高。举办首届戏曲文化节，成功创建“全民悦读咸宁朗读示范基地”“湖北楹联文化城市”，被命名为“中国古瑶文化之乡”。稳妥处置各类突发事件，推进“一感两度”提升工程，嘉鱼县荣获全国综治最高奖“长安杯”。加强食品药品安全监管，保持安全生产形势平稳，社会大局和谐稳定。

（七）聚力作风转变提高行政效率。认真学习贯彻党的十九大精神，扎实开展“两学一做”学习教育、“支部主题党日”“走村巷?连民心”活动，深入推进政府系统作风建设。加强行政监察和审计监督，严肃查处各类违纪违法案件。全面建立权责清单制度，法治政府建设顺利推进，行政效率明显提高。认真执行市人大及其常委会制定的地方性法规和决议、决定，自觉接受市人大及其常委会法律监督和工作监督，主动接受市政协民主监督和社会舆论监督，按期办复人大代表议案、建议127件，政协委员提案124件，办复率100%，满意率98%。

双拥创建、国防动员和后备力量建设进一步加强。援疆工作深入推进。妇女儿童、青少年、残疾人等事业全面发展。科普、保密、档案、方志、统计、物价、水文、气象、人防、民族宗教、防震减灾、社科、政府咨询等工作取得新成绩。

各位代表，过去的一年，我们跨进了新时代，凝聚了新能量，开启了新征程。一年的拼搏汗水，已汇聚成咸宁改革发展的强大动力。成绩来之不易，这是省委、省政府和市委正确领导的结果，是市人大、市政协监督支持的结果，是全市人民奋勇拼搏的结果。在此，我谨代表市政府，向全市广大干部群众，向各位人大代表、政协委员和各民主党派、工商联、无党派人士，向离退休老同志，向驻咸解放军、武警消防官兵、公安干警，向中直、省直驻咸和在咸投资兴业的企业家，向所有关心支持咸宁发展的社会各界人士和海内外朋友，致以崇高的敬意和衷心的感谢！

一年的发展，我们深深体会到：必须坚持党对经济工作的领导，保证经济沿着正确方向发展；必须坚持以人民为中心的发展思想，让全体人民在共建共享发展中有更多获得感；必须坚持以经济建设为中心，始终把发展作为解决一切问题的基础和关键；必须坚持新发展理念，持续推进绿色崛起不动摇；必须坚持改革创新，最大限度激发人民群众的创造活力；必须坚持务实重行，坚决做到马上就办、真抓实干。

同时，我们也清醒地认识到，发展不够仍然是咸宁最大的实际、最大的市情。存在的主要问题是：经济规模不大，总体实力不强；产业结构不优，发展质效不高；创新动力不足，科技支撑不强；对外开放不够，经济外向度不高；民生领域短板较多，脱贫攻坚任务艰巨；少数干部担当意识不强，推动发展的能力不足等等。需要特别说明的是，地区生产总值、城镇常住居民人均可支配收入增速未实现预期目标。我们将正视问题，切实加以解决。

二、2018年工作思路和主要任务

2018年是贯彻党的十九大精神的开局之年，是改革开放40周年，是决胜全面建成小康社会、实施“十三五”规划承上启下的关键一年。综观国内外大势，世界经济逐步回暖，我国经济稳中向好，湖北发展势头强劲。经过多年发展，我市发展条件更加有利，内在优势更加彰显，向好趋势更加巩固，仍处于可以大有作为的重要战略机遇期。同时，我市经济处在总量攀升期、工业化中期、投资拉动期、动能转换期、爬坡过坎期，面临做大总量、调优结构、提升质量“三大任务”的重大挑战。我们既要看清有利形势，又要勇于面对挑战，抢抓机遇，争先进位，以永不懈怠的精神状态和一往无前的奋斗姿态，创造无愧于历史的光辉业绩，书写新时代咸宁发展的崭新篇章！

2018年经济社会发展的基本思路：全面贯彻党的十九大精神，以习近平新时代中国特色社会主义思想为指导，认真落实中央、省委和市委经济工作会议精神，坚持发展第一要务，坚持稳中求进工作总基调，坚持新发展理念，紧扣我国社会主要矛盾变化，按照高质量发展的要求，以供给侧结构性改革为主线，以建设现代化经济体系为目标，深入实施“创新驱动、绿色崛起”战略，坚决打好“三大攻坚战”，决胜全面建成小康社会，率先建成全省特色产业增长极，全力打造中国中部“绿心”和国际生态城市，建优建美香城泉都，开启新时代咸宁社会主义现代化建设新征程。

2018年经济社会发展的主要目标：地区生产总值增长8%左右；规模以上工业增加值增长8%以上；固定资产投资增长12%；一般公共预算收入增长9%；社会消费品零售总额增长11.5%；出口总额增长10%；居民消费价格涨幅控制在3.5%以内；城乡居民收入增长与经济增长同步；城镇登记失业率控制在4.5%以内；完成省定节能减排任务。

实现上述目标，要着重抓好以下七个方面工作：

（一）坚持发展第一要务，推动经济持续健康发展。发展是咸宁最紧迫的任务。必须一心一意谋发展，聚精会神搞建设，做大总量，优化结构，提升质量，进一步夯实发展底盘，使发展成为咸宁最强的声音、最美的乐章！

积极扩大有效投资。发挥有效投资的关键性作用，深入推进“三抓一优”。强化项目投资。扎实开展“一季一拉练、一季一开工”等活动，突出抓好省领导督办重大产业项目、省级重点项目、10亿元以上新开工项目、100个市级重点项目，烧旺项目投资之火。突出招商引资。落实招商引资“17条”，全年引进国内外500强企业投资项目3个以上、10亿元以上项目10个、亿元以上项目100个，招商引资到位资金超过1000亿元。围绕产业链精准招商，加大总部经济引进力度，着力提高项目落地率和开工率。积极向上争资。出台争资考核办法，力争更多项目挤进国家和省“笼子”，咸宁元素和咸宁份额大幅增加。激活民间投资。进一步放开准入领域，大力推进PPP模式，加快建成一批示范带动项目。优化环境护资。继续实施重大项目建设考核、领导领衔包保、项目推进“五个一”制度，落实工业项目“零”审批政策，构建“亲”“清”政商关系，营造重商亲商的投资环境。

持续释放消费潜力。发挥消费的基础性作用，全力激活消费市场。继续保持建筑及装潢材料类、家具类、电器类等快速增长势头，大力提升房地产、汽车等改善性消费，积极发展健康、文化、养老、体育等服务消费，不断扩大智能、绿色、信息、时尚等新兴消费，打造多点支撑的消费增长格局。积极开展消费促进活动，构建便民生活服务体系。实施“荆楚优品”咸宁工程，培育一批省级以上名企名牌名品。实施质量强市战略，打造“咸宁标准”。加强诚信建设，营造良好市场环境。

加快促进外贸增长。发挥外贸的促进性作用，提升经济外向度。实施“一企一策”，重点培育瀛通、东森等龙头企业。实施外贸转型升级工程，利用境内外展会、电子商务、境外营销点等开拓国际市场。优化出口商品结构，支持名特优产品出口。坚持走出去和引进来并重，支持企业到境外承包工程和开展境外投资，扎实开展对外项目合作，重点引进农产品生产、外销企业，不断提高外资项目落地率、合同利用外资转化率。

（二）大力振兴实体经济，加快构建现代产业体系。实体经济是现代产业体系的主体，现代产业体系是现代化经济体系的重要支撑。实体兴，则产业兴；产业强，则经济强。必须竭尽全力抓产业，使产业成为咸宁的财富之本、人民的幸福之源！

构建现代工业产业体系。坚持一手抓传统产业优化升级，一手抓新兴产业培育，推动“五大千亿产业”发展。加强园区建设，完善产业规划和功能分区，促进“一区四园”协调发展，深度推进军民融合产业园建设。加快产业发展，实施“双十”工程，培育10个三百亿重点成长型产业集群，10家产值50亿元引导示范性的关键企业。发展壮大企业，实施中小企业成长工程，全年新增入规企业75家，力争两年规上工业企业净增120家、总数突破1000家。推进质量建设，实施产业质量提升工程，完善重点技改项目储备库，精准对接亿元以上技术改造项目。推进产业创新，实施科技创新工程，培育一批智能制造、新型生产模式企业，科技小巨人和隐形冠军企业达到30家。

构建现代农业产业体系。坚持质量兴农、绿色兴农，深化农业供给侧结构性改革，提质传统农业，做强农产品加工业，壮大新产业、新业态，提高农业综合效益。推进农业专业化、标准化、规模化、市场化生产，加快打造一批田园综合体。新建农业标准化生产基地10万亩，抓好3个省级现代农业产业园建设。实施优质粮油工程，推动粮油经济发展。加快“三品一标”建设，新增20个品牌。大力推进农业产业化，新增国家级龙头企业1家，实现农产品加工业产值率先突破千亿。新增“农业+休闲”“农业+旅游”“农业+文化”“农业+科技”示范基地各30个。实施农业信息技术进村入户工程，推广可视农业、农业互联网+。加快林业产业发展，实现产值200亿元。

构建现代服务业产业体系。以“大旅游、大健康、大文化”为主体，以金融和物流为支撑，构建现代服务业发展格局。加快发展大旅游，积极创建国家全域旅游示范区，推进131军旅小镇、黄龙山风景区、龙泉山万亩野樱花、富水湖旅游开发等项目建设。加快发展大健康，全面实施“养在咸宁”工程，推进梓山湖大健康产业示范区、金色年华养生谷、药姑山健康产业园等项目建设。加快发展大文化，实施文化项目带动工程，推进田野国家乡村公园、世界茶叶第一古镇、闯王文化园等项目建设，文化产业增加值占GDP比重超过3%。大力发展现代金融，推进绿色金融、科技金融、普惠金融，发挥产业基金的作用，开展大型银企信贷对接活动，推进企业上市挂牌，增强服务实体经济能力。贺胜金融小镇引进基金公司达到200家，规模达到500亿元。大力发展现代物流，高标准规划咸宁现代物流园，推进捷利泉都现代物流产业园、湘鄂赣边贸市场等项目建设。积极发展大数据产业、科技服务业、商务服务业，促进电子商务、邮政业和平台经济发展。

构建现代城镇产业体系。以城镇化为抓手，推动人口与产业共生，推进城镇产业体系建设。实施“十大规划行动”，推进城市功能新区、棚户区改造、特色小镇建设，以新型城镇化拉动投资和消费。推进产业园区与城镇融合发展，以产兴城、产城互动，支持建筑业、餐饮业做大做强，发展装潢、家具、环保等产业。加快建设以完善城市功能为主的产业体系，推进道路交通、垃圾处理、照明监控、供排水等工程建设，加快金融保险、城市设计、创意文化、人力资本服务等产业发展。按照“一村一品、一镇一业”的基本要求，重点打造20个市级以上特色产业小镇。

构建现代绿色产业体系。加快形成绿色生产和生活方式，建立绿色低碳循环发展的经济体系。大力发展节能环保产业、清洁生产产业、清洁能源产业，加快建设循环经济园区、低碳产业园区，支持通山做大做强清洁能源产业群。推进咸宁高新区绿色创新体系建设，打造食品饮料、新能源新材料、生物医药、电子信息、先进装备制造等绿色产业集群。建设国家农业可持续发展试验示范区暨农业绿色发展试点先行区，打造“一区六园十八景”。推进省级农产品优势区建设，支持赤壁建设绿色生态农产品交易基地，推出一批绿色农业品牌。加快发展低碳环保服务业，催生一批绿色经济。

（三）激发创新创业活力，加快创新咸宁建设步伐。创新是第一动力，人才是第一资源。必须坚定不移地实施创新驱动和人才强市战略，将咸宁建设成为人才的聚集地、创新的试验田，让创新源泉不断涌流、人才活力不断迸发！

实施人才强市战略。推动事业留人、感情留人、待遇留人，聚天下英才而用之，打造人才高地。创新方式引才。实施“五个引才”工程，全力引进一批海内外科技领军人才、管理人才、高技能人才和优秀青年人才。搭建平台用才。实施 “百千万”人才工程、“科技智囊团”一线人才工程，开展“院士专家咸宁行”等活动，加强人才项目库建设，推进乡土人才建设，最大限度激发人才创造活力。探索建立人才“旋转门”机制，赋予用人主体更大自主权。优化服务留才。出台人才优惠政策，妥善解决人才在项目建设、居留落户、子女就读等方面后顾之忧，增强人才的获得感和归属感。

扎实推进科技创新。继续实施科技创新“五大工程”，力争专利申请量突破2000件、高新技术企业达到220家、高新技术产业增加值占GDP比重突破10%。突出咸宁高新区创新龙头地位，力争把光谷南科技城打造成国家级科技孵化器、晶津城和启迪之星打造成省级科技孵化器。新建众创空间6家，新增入孵科技企业100家。加快企业创新主体建设，发挥湖北科技学院、咸宁职院、咸宁职教集团等创新领头作用。探索建立科研经费使用管理新机制，推动科技成果转化。新建省级技术创新平台2家，推进省级智能机电产业技术研究院建设。

全面推进创新创业。重点打造“梦香城?咸宁市人才创新创业超市”，建设市、县整体联动的青年创业孵化器平台。发挥“咸商”等桥梁纽带作用，开展“迎老乡、回故乡、建家乡”活动，吸引咸宁籍人士返乡创业。开展“创新创业大讲堂”，举办第二届“南鄂杯”全球高层次人才创新创业大赛，培育壮大创客群体。推进技能强市战略，办好第四届咸宁市职业技能大赛。加强科技金融融合，搭建创新创业融资平台。弘扬劳模精神和工匠精神，激发和保护企业家精神，营造崇尚创新创业的浓厚氛围。

（四）抢抓重大战略机遇，更大力度推进改革开放。改革开放是咸宁最强大的动力，必须发挥改革的牵引作用、开放的先导作用，以舍我其谁的气势，敢打拦路虎，勇啃硬骨头，向着改革目标奋勇前进！

深入推进供给侧结构性改革。深入实施淘汰落后产能、违法违规项目清理、联合执法三项行动，全部退出煤炭等落后产能。推进租购并举，发展住房租赁市场，确保商品房去库存消化周期控制在10个月左右。完成棚户区改造9642套。改善城市用地供给结构，着力打造梓山湖?悦水湾、咸宁高铁商务区、金融信息港等城市综合体。发行企业债20亿元，保障重大项目建设的资金需求。防范和打击非法集资，强化互联网金融风险专项整治，加强政府性债务管理。清理涉企收费，整治乱收费，确保降低企业成本10亿元以上。统筹推进补短板三年十大重点工程。

加快推进重点领域改革。深化行政审批制度改革，建立覆盖市县乡三级标准管理体系。实施市场准入负面清单制度改革试点，完善“证照分离”“多证合一”改革。深化综合行政执法体制改革，实现“双随机一公开”监管全覆盖，全面实施公平竞争审查制度。推进政务服务提升行动，建设数据共享服务平台。推进“多规合一”，强化规划引领作用。全面推行零基预算改革，加强预算绩效管理。推进市县乡三级财政事权与支出责任划分改革。深化国地税征管体制改革。加快投资项目网上在线审批监管平台应用。深入推进国资国企改革，加快“三供一业”分离移交。

全面提升对外开放水平。大力推动武咸一体化，建设北部空间，实现交通一体、产业对接、市场互融、服务共享。抢抓长江岸线资源整合机遇，推动规划修编和项目实施，加快潘家湾物流园区建设。支持通城、赤壁、崇阳与毗邻地区在基础设施、市场建设、公共服务等方面加强合作，扩大与长江沿线城市合作范围。主动参与“一带一路”建设和中俄“两江地区”合作项目，深度推进与蒙古、古巴及中亚各国合作。加强与韩国太白市和义王市、法国奥贝维利耶市等国际友城经济文化交流。

（五）实施区域发展战略，统筹推进城乡融合发展。发展不平衡不充分是咸宁突出的矛盾。必须以更大的力度、更有效的举措，推进区域协调共进、城乡融合发展，确保小康路上不落一人！

提升中心城区品质。实施城市“双修”工程，加快新一轮城市总体规划修订，高标准推进“一城十区二十景”建设，擦亮“香城泉都”品牌。实施基础设施建设工程，加快推进107国道咸安绕城段、咸宁大道西延伸等重点项目建设。实施城市功能提升工程，推进淦河流域综合整治、大洲湖生态治理等工程，加快地下综合管廊、城市防洪排涝、黑臭水体治理和“海绵城市”建设。实施公共服务提升工程，配套完善学校、医院、农贸市场、公共停车场等，加快永安阁、图书馆、档案馆、体育馆、工人文化宫等项目建设，推进街景整治、公园提升和美化亮化工程，优化完善充电站场等设置。

实施乡村振兴战略。实施“市民下乡、能人回乡、企业兴乡工程”，推进城乡资源要素合理流动和优化配置。推进农村集体产权制度改革，完成203个试点村改革任务。发展适度规模经营，全市土地流转面积达到70万亩以上。培育新型经营主体，新发展市级以上示范家庭农场40家。抓好防洪抗旱、农田排灌、水毁修复等9大方面工程建设，提高农业综合生产能力。大力推进农村“双治”“厕所革命”“扫帚革命”，强化农村建房管控，建设30个高标准美丽示范乡村。积极探索农村公共设施社会化建管模式，推进“四好农村路”建设。健全自治、法治、德治相结合的乡村治理体系，建设一支懂农业、爱农村、爱农民的“三农”工作队伍。

推动区域协调发展。完善区域交通网络，扎实做好咸宁机场前期工作，加快嘉鱼长江大桥、武深高速嘉鱼北段等建设步伐，推进赤壁长江大桥开工建设，全力争取咸宁（通山）至九江（永武）高速、通城至修水高速、城区南外环高速等列入中期规划，积极做好武幕扶贫旅游公路论证工作。高昂市区龙头，以咸宁高新区为主战场，推进市区一体发展，做大做强市域经济。加快咸嘉生态文化城镇带和幕阜山绿色产业带建设，协调推进沿线招商引资、产业发展和特色小镇建设。积极谋划“两湖”流域等区域性生态保护和绿色发展。振兴县域经济，支持县（市、区）按照各自定位，争创全省新的经济增长点。加快农业转移人口市民化，全市城镇化率提高1.2个百分点。

（六）建优建强绿色品牌，打造山清水秀美丽咸宁。绿色是咸宁最靓丽的名片。必须坚持人与自然和谐共生，在绿水青山中打造金山银山，擦亮绿色品牌，建设美丽咸宁，使咸宁人民享受永久的绿色福利！

着力解决突出环境问题。深入开展“三非三水”整治工作，持续开展长江大保护十大行动。严格跨界断面水质考核，全面落实河湖库长制，加快推进斧头湖、富水湖、黄盖湖、陆水河流域综合整治，确保57个乡镇污水处理厂全部建成。突出抓好大气污染联防联控联治，打赢蓝天保卫战，确保空气质量优良率在78%以上，PM10浓度均值控制在每立方70微克以下，空气质量综合指数稳居全省前列。扎实开展化肥农药“两精两减一增效”行动，全面加强农业面源污染整治，确保42个乡镇垃圾压缩中转站建成投运。以钉钉子精神抓好中央环境保护督察反馈意见整改落实。

实施生态保护修复工程。积极推进国家生态文明示范市、国家生态保护与建设示范区建设，深入开展森林城镇和绿色示范乡村创建工作，力争创建2个省级生态文明建设示范县、4个省级森林城镇、50个绿色示范乡村。加快高标准农田、占补项目和生态土地整治试点项目建设，努力提高新增耕地质量。严守永久基本农田红线。持续开展自然保护区“绿盾”行动，实施幕阜山森林质量提升工程，加快推进矿山“复绿”工程。实施国土绿化精准灭荒三年行动计划，完成年度荒山造林15.4万亩。引导社会各方面资金参与生态保护和修复工程，培育一批从事生态保护修复的专业化企业。完成生态保护红线划定工作。

加强生态文明制度建设。严格落实生态环保“党政同责、一岗双责”，全面实行领导干部自然资源资产离任审计，严格执行生态环境损害责任终身追究制。健全环保监管体系，落实省以下环保机构监测监察执法垂直管理制度改革，完善生产者责任延伸制度。健全完善生态环保市场化制度，探索建立生态补偿机制，推行环境污染第三方治理。加快构建政府主导、企业主体、社会组织和公众共同参与的环境治理体系，凝聚全社会共建美丽咸宁的强大合力。

（七）坚持人民利益至上，满足人民美好生活需要。民心是最大的政治，民生连着民心。必须坚持以人民为中心的发展思想，把人民群众的小事当作政府工作的大事，一件接着一件办，一年接着一年干！

坚决打好脱贫攻坚战。按照精准脱贫总要求，突出产业就业扶贫，重点发展十大扶贫产业。创新利益联结机制，加快专业合作社建设，推动更多贫困户纳入产业链条。全面推进健康扶贫工程，严格落实补充医疗保险和民政医疗救助政策。整合扶贫资金和项目，加大小额扶贫贷款力度，向特定贫困群众精准帮扶，向深度贫困村聚焦发力。开展扶志、扶智活动，激发贫困户内生动力。强化驻村帮扶工作，加大扶贫督查考核力度。加强贫困对象的动态管理，确保67178人脱贫、48个贫困村出列、崇阳县和通山县“摘帽”。

加强就业和社会保障工作。统筹推进高校毕业生、农民工和困难群体就业创业，新增城镇就业3.7万人。提高退休人员基本养老金，全额资助特殊困难群体参加居民养老保险、医疗保险。推进社保扩面征缴工作，夯实缴费基数，提高参保率和缴费率。落实降低企业社保费率政策，健全待遇正常调整机制。完善工资收入结构，保持物价总体稳定，努力提高城乡居民收入水平。提高低保保障标准，完善医疗救助、临时救助、慈善求助体系。研究出台物业管理实施办法。抓好农村饮水安全巩固提升工程。实施健康促进和留守儿童关爱行动，保障妇女和未成年人合法权益，支持发展残疾人事业。

促进社会事业全面进步。优先发展教育事业，加强学前教育，推进城乡义务教育一体化，实施主城区教育三年行动计划。全市新建、改扩建学校131所，着力解决入园难、入园贵和择校热、大班额问题。健全学生资助政策，实现家庭经济困难学生资助全覆盖。深化医药卫生体制改革，推进分级诊疗和“三医联动”，实施统一的城乡居民基本医保制度，推动跨省异地就医联网结算。推广家庭医生签约服务。全面提升乡镇卫生院服务能力，加强乡村医生队伍建设。健全食品药品安全监管体系，全力创建国家食品安全示范城市。继续举办咸宁国际温泉马拉松，打造品牌体育赛事。加强城乡公共健身场所建设，打造居民15分钟健身圈。完善计划生育服务管理，促进人口均衡发展。加快发展健康养老事业，推进医养结合。推动全民阅读，打造“书香咸宁”。践行社会主义核心价值观，努力营造向上向善、积极进取的人文氛围。加快县市区“四馆三场”建设，推进公共文化服务标准化、均等化。加大地方特色文化的挖掘和宣传力度，继续做好赤壁市羊楼洞、新店明清石板街申遗工作。认真做好档案和编史修志工作。

推动社会治理共建共治共享。加强社会心理服务体系、社区治理体系建设，妥善化解信访矛盾和历史遗留问题。构建公共法律服务体系，提高全民法律素质。推进信用体系建设，确保创建国家社会信用体系示范城市通过国家评估验收。保障农民工工资支付，构建和谐劳动关系。健全立体化社会治安防控体系，推进“雪亮工程”和数据整合，开展破案攻坚系列行动，严厉打击网上虚假信息诈骗、倒卖个人信息等违法行为。压实安全生产责任，开展“打非治违”专项行动，坚决守住安全生产红线。继续做好国防动员、国家安全、民族宗教、人民防空、双拥共建等工作，加强智库建设，支持群团组织发挥更大作用。

三、强化政府自身建设，打造人民满意政府

新时代要有新气象，更要有新作为。必须加强政府自身建设，履职尽责，善作善成，全面提升服务人民群众的能力。

突出政治建设。牢固树立“四个意识”，始终同以习近平同志为核心的党中央保持高度一致。认真学习习近平新时代中国特色社会主义思想，扎实开展“不忘初心，牢记使命”主题教育。坚决贯彻落实上级党委政府的重大决策部署，确保政令畅通、执行有力。坚决在市委领导下开展工作，确保市委的决策部署在政府工作中得到全面贯彻落实。

严格依法行政。全面贯彻法治政府建设实施纲要，落实宪法宣誓制度，增强公务人员法治意识。开展行政执法突出问题专项治理，进一步营造良好执法环境。推进政务公开，加强政策宣传解读，增强决策透明度。自觉接受人大、政协和人民群众的监督，认真办理人大议案、建议和政协提案，主动听取人大代表、政协委员意见和建议，推进科学决策、民主决策、依法决策。

坚持真抓实干。说实话、谋实事、出实招、求实效，把雷厉风行和久久为功有机结合起来。用数据决策、用数据说话、用数据检验，反对空话假话套话。把发展目标和任务项目化，用项目化思维和办法推进工作。做到知行合一，说必干，干必成。加大检查督办力度，为干事创业者撑腰鼓劲，对落实不力者严格问责。

加强能力建设。建设学习型政府，加强学习培训，注重培养干部专业能力、专业精神。增强干部改革创新、科学发展、依法行政、群众工作、狠抓落实、驾驭风险的本领，提高适应新时代新要求的能力。推行一线工作法，鼓励干部到招商引资、项目建设、维稳处难、脱贫攻坚一线进行锤炼，提升能力，展现作为。

打造清廉政府。严格执行中央关于作风建设新要求，落实全面从严治党主体责任和“一岗双责”，坚决抵制新形势下形式主义、官僚主义。树立过紧日子的思想，强化预算刚性约束。加强审计监督，对重要领域、重点单位、关键岗位进行常态化监督。持续正风肃纪，加强廉政教育，以零容忍态度惩治腐败。

各位代表，新思想引领新航程，新时代创造新辉煌。让我们紧密团结在以习近平同志为核心的党中央周围，在省委、省政府和市委的坚强领导下，不忘初心，牢记使命，埋头苦干，奋力争先，为决胜全面建成小康社会、率先建成全省特色产业增长极、打造中国中部“绿心”和国际生态城市、建优建美香城泉都、全面开启新时代咸宁社会主义现代化建设新征程而努力奋斗！
